# A multi-scale parallel weighted fusion dynamic attention method for citrus leaf disease recognitions

**DOI:** 10.3389/fpls.2026.1783499

**Published:** 2026-04-30

**Authors:** Baijing Wu, Ke Gao, Jiren Gu, Guanghui Yan

**Affiliations:** 1 School of Electronic and Information Engineering, Lanzhou Jiaotong University, Lanzhou, China; 2 Jiujiang Polytechnic University of Science and Technology, Jiujiang, China

**Keywords:** citrus management, disease detection, dynamic sparse attention, RT-DETR, smart agriculture

## Abstract

To address the low detection accuracy caused by leaf occlusion, the loss of disease targets, and complex backgrounds in citrus leaf disease detection, this study proposes a leaf disease detection method termed DBG-DETR (a real-time detection transformer with DMGF, BDFF, and GSDT). Firstly, a DMGF-ResNet18 (dynamic multi-scale gating fusion block) is designed as the disease feature extraction module. By leveraging multiscale parallel depthwise separable convolutions, this module adaptively extracts and fuses rich disease-related features. Secondly, a GSDT (gated sparse dynamic transformer) is introduced to focus on deep features. Through a dynamic gating mechanism and Top-K sparse attention, GSDT reduces model parameters while enabling the network to concentrate on disease regions. Finally, a BDFF (bi-directional dense feature fusion module) is proposed to facilitate effective interaction between shallow and deep features, achieving efficient disease feature fusion. Experimental results on a real or chard dataset demonstrate that, compared with the baseline model, DBG-DETR improves P, mAP mmAP, R and F1 by 3.31%, 3.40%, 4.11%, 3.89% and 3.59%, respectively, while reducing the number of parameters by 3.78 MB. These results indicate that the proposed method significantly enhances disease detection performance in complex background environments and provides reliable technical support for intelligent citrus orchard management.

## 1 Introduction

Navel orange is one of the most widely cultivated citrus varieties in China, characterized by the largest planting area, the highest brand value, and the most extensive market scale within the citrus industry. According to the national citrus industry technology system plan and data from the China Rural Statistical Yearbook, by 2023, the cultivation area of navel oranges in China had exceeded 15 million mu, with an annual output surpassing 18 million tons, accounting for nearly 40% of the total citrus production nationwide (Fan and Zhang, 2004). Among major producing regions, Gannan ranks first nationwide in fruit industry output value. In 2023, the output value of the navel orange industry in this region reached 69.127 billion CNY, making it a pillar industry for rural revitalization in former revolutionary base areas. Despite the rapid growth of the citrus and navel orange industries, they continue to face persistent threats from bacterial diseases such as huanglongbing (HLB). These diseases often manifest as symptoms including black spots, canker lesions, greening, and melanosis on citrus leaves, which significantly degrade fruit quality and reduce yield. If not detected and managed in a timely manner, such diseases can spread across multiple generations of plants, making them extremely difficult to eradicate. Therefore, the rapid and accurate identification of citrus leaf diseases is of critical importance for effective pest and disease control and for ensuring the sustainable and healthy development of the citrus industry, with long-term and far-reaching implications (Jrondi et al., 2024; Injante and Sánchez Isuiza, 2025).

In recent years, advances in emerging digital technologies, including the Internet of Things (IoT), big data analytics, and artificial intelligence, have accelerated the transition of agriculture toward data-driven and intelligent paradigms (Azadi et al., 2021). Within this technological shift, artificial intelligence has played a pivotal role by enabling high-capacity processing and analysis of complex agricultural data, particularly in visual perception, information extraction, and predictive analysis. These capabilities have substantially enhanced precision management and evidence-based decision making across agricultural production processes, offering a viable technological foundation for the modernization of contemporary agriculture (Upadhyay et al., 2025). The citrus industry, distinguished by its mature production system, significant economic contribution, and strong demand for intelligent solutions, has become a representative domain for the deployment and validation of smart agriculture technologies (Jafar et al., 2024). Among various management tasks in intelligent citrus production, the automated detection of plant diseases with high accuracy and efficiency is essential for maintaining production stability and economic sustainability. Nevertheless, conventional field inspection practices rely heavily on manual observation, which is often constrained by labor intensity, subjective bias, and limited timeliness, making them unsuitable for large-scale orchards requiring early warning and precise disease intervention. Consequently, computer vision–driven disease detection approaches grounded in deep learning have emerged as an effective alternative. These methods are capable of learning discriminative representations from complex visual environments and performing real-time inference in an end-to-end manner. By training deep neural networks, such as convolutional architectures, citrus leaf images can be automatically analyzed to identify disease symptoms, thereby establishing a robust technical basis for intelligent, noninvasive monitoring and management of citrus diseases.

In summary, the integration of artificial intelligence techniques and deep learning models into complex agricultural scenarios, represented by the citrus industry, has substantially advanced the automation and intelligence of disease recognition, demonstrating considerable technological potential and industrial value (Lanjewar and Parab, 2023). Nevertheless, despite the progress achieved by existing studies, significant limitations remain when these methods are deployed in real world cultivation environments. First, most current models are trained on idealized datasets with relatively simple backgrounds, which makes them poorly suited to the diverse conditions encountered in practical orchards, such as varying illumination, severe leaf occlusion, and heterogeneous disease appearances. As a result, their robustness and generalization capability in real planting environments are often limited. In addition, the acquisition and annotation of disease images in orchards are labor-intensive and costly, and the scarcity of large-scale, high-quality public datasets further restricts model optimization and performance improvement. Consequently, overcoming the combined challenges posed by data scarcity and environmental complexity, and achieving accurate and reliable leaf disease recognition and monitoring under real world conditions, remains a critical and unresolved issue in the field of intelligent agricultural disease detection. To address these challenges, this paper proposes a lightweight leaf disease detection method DBG-DETR, tailored for citrus cultivation environments. The proposed approach effectively reduces model complexity and parameter scale while maintaining high detection accuracy and robustness in complex backgrounds. The main contributions of this study are summarized as follows:

1. A dynamic multi-scale gating fusion (DMGF) backbone feature extraction network is proposed. The DMGF blocks are embedded into the four C2f stages of the ResNet18 backbone. By adopting parallel depthwise separable convolutions, the network separately extracts longitudinal, transverse, and local features of leaf diseases. An adaptive weighted fusion strategy is then applied to integrate these features, which effectively enhances the representation of small-scale and occluded leaf disease targets while ensuring computational efficiency, enabling the network to capture richer disease features.
2. To address the difficulty of accurately distinguishing disease features from background interference in complex scenes, a gated sparse dynamic transformer (GSDT) module is introduced. Linear additive operations are employed to replace conventional matrix multiplication, thereby reducing computational overhead. Combined with a dynamic gating mechanism and Top-K sparse attention, the proposed module enables the network to adaptively focus on critical disease regions, effectively improving disease discrimination performance under complex background conditions.
3. To mitigate the loss of fine-grained leaf disease features during feature sampling in complex environments, a bidirectional dense feature fusion (BDFF) module is proposed. Shallow and deep features are aligned through downsampling, and a modulation fusion module (MFM) is incorporated to adaptively facilitate cross-level feature interaction and fusion. This design enhances the model’s ability to represent occlusion, illumination variation, and diverse disease morphologies, significantly improving the localization accuracy of disease regions.

## 2 Related works

### 2.1 Image-based agricultural disease detection

Research on image based plant disease detection has gradually evolved from early traditional recognition approaches relying on hand crafted features to a data driven stage of intelligent perception. Early studies primarily employed manually designed features and classification models pretrained on large scale generic datasets to achieve high accuracy disease recognition on single leaf images under relatively controlled laboratory conditions (Khattak et al., 2021). To improve practical applicability in real world scenarios, subsequent studies have increasingly focused on disease detection in complex orchard environments. By introducing deep learning–based object detection or semantic segmentation models, these approaches enable precise localization and analysis of disease regions in natural scenes (Qi et al., 2022). incorporated a squeeze and excitation (SE) attention module into the YOLOv5 (you only look once) network, enhancing the model’s ability to extract tomato disease features and thereby improving detection accuracy (Gülmez, 2025). systematically reviewed the application of ResNet (residual network), VGG (visual geometry group), and MobileNet in potato disease detection, analyzing the strengths and limitations of different models and providing valuable insights for deep learning–based agricultural disease research. Such studies contribute to improving disease recognition accuracy, optimizing field management strategies, and supporting sustainable enhancements in crop productivity and food security. Furthermore (Lu et al., 2024), proposed an intelligent disease detection and question answering system that integrates Transformer based architectures with multimodal agricultural data, including images, textual information, and sensor data, to support disease identification and agricultural decision making (Wang et al., 2024). developed a rice disease detection model that combines a deep convolutional generative adversarial network (DC-GAN) with a multidimensional feature compensation residual network (MDFC-ResNet), achieving effective disease detection and early warning. In addition (Wang and Liu, 2024), proposed a tomato disease detection method that integrates attention mechanisms with a dynamic feature fusion network, significantly improving the detection accuracy and robustness of small disease targets in complex environments (Somkunwar et al., 2024). employed convolutional neural networks (CNNs) to predict and optimize resource allocation in agricultural production (Qu and Su, 2024). provided a comprehensive review of the advantages of various deep learning methods in weed recognition, demonstrating that the integration of state-of-the-art techniques with agricultural practices, supported by intelligent devices, offers a promising pathway toward efficient and environmentally friendly weed management in modern agriculture.

Existing studies have achieved notable progress in crop specific scenarios, effectively improving detection accuracy and scene adaptability. However, in practical agricultural applications and deployment settings, these methods still suffer from relatively large model sizes, which limit their ability to process video data in real time. Moreover, in real world citrus orchard environments characterized by dense leaf overlap and strong background interference, the robustness of existing models remains limited. To address these challenges, this study proposes DMGF module enhances feature sensitivity and localization accuracy for small-scale and occluded disease targets, thereby enabling high precision real-time detection of citrus leaf diseases in complex orchard environments.

### 2.2 Citrus leaf disease detection

In intelligent disease detection research for citrus crops, the identification of key diseases such as huanglongbing, citrus canker, and scab primarily relies on leaf level manifestations. To improve the performance of leaf disease detection, extensive studies have been conducted from multiple perspectives, including model architecture design, data augmentation strategies, and multimodal feature fusion (Zheng et al., 2025). optimized the YOLOv8n model by introducing adaptive convolution to dynamically enhance disease-feature responses and by improving the C2f layers with fast convolution, thereby improving recognition accuracy and efficiency without incurring additional computational overhead while also expanding disease category coverage. However, its ability to focus on discriminative features under complex background interference still requires further validation (Barman et al., 2020). designed a lightweight convolutional neural network that achieved a favorable balance between accuracy and inference speed for mobile deployment scenarios. However, it remains insufficiently sensitive to small-scale lesions or subtle early-stage symptoms (Çetiner, 2022). developed a classification model capable of effectively distinguishing black spot disease, citrus canker, and huanglongbing, achieving satisfactory classification performance. However, the model contains a relatively large number of parameters, which limits its deployment efficiency on edge devices (Storey et al., 2022). applied Mask RCNN to apple rust disease segmentation, demonstrating clear advantages in small-target disease recognition (Huangfu et al., 2024). employed an improved lightweight HHS-RTDETR model—incorporating hybrid wavelet directional filter banks and a high-level screening feature pyramid network—for huanglongbing detection in citrus, achieving a detection accuracy of 92.4%, which outperformed commonly used models such as YOLOv5m and YOLOv8n. However, the robustness and generalization capability of this approach remain limited under complex orchard environments.

In summary, although citrus disease detection has achieved stage wise progress in model optimization, lightweight deployment, and classification accuracy, existing approaches still exhibit notable limitations when applied to complex real world navel orange orchard environments, particularly in terms of generalization capability, adaptability to small scale and occluded targets, and real-time performance in dynamic inspection systems. To address these challenges, this study proposes BDFF module, which enhances the model’s ability to recognize overlapping, occluded, and small disease targets by strengthening cross-level feature interactions and selectively fusing multi-level representations.

## 3 DBG-DETR

The RT-DETR (Zhao et al., 2023) model mainly consists of a backbone, an adaptive intra-scale feature interaction (AIFI) module, and a cross-scale feature fusion module (CCFM), and has demonstrated certain advantages in citrus leaf disease detection. The backbone is built upon ResNet18, which is capable of extracting rich multi-level disease features from input images. The AIFI module enhances feature representation by employing a residual scale self-attention mechanism, enabling the model to focus on disease related information in occluded regions under complex backgrounds and thereby improving its ability to capture critical features. The CCFM module integrates multi-scale features through a progressive fusion strategy, which improves the recognition and discrimination of small-scale and early-stage disease lesions. However, in real world citrus orchard environments characterized by severe occlusion and small disease targets, these modules rely heavily on multiple convolutional operations, which can progressively weaken the representation of small-target disease features during feature sampling, causing such features to become blurred or even lost in deeper network layers. In addition, all three modules employ pooling layers to reduce computational complexity. The local extremum selection mechanism of pooling layers tends to overlook subtle features of small disease targets, leading to the loss of critical information and increasing the risk of missed detections and false positives.

**Figure 1:**
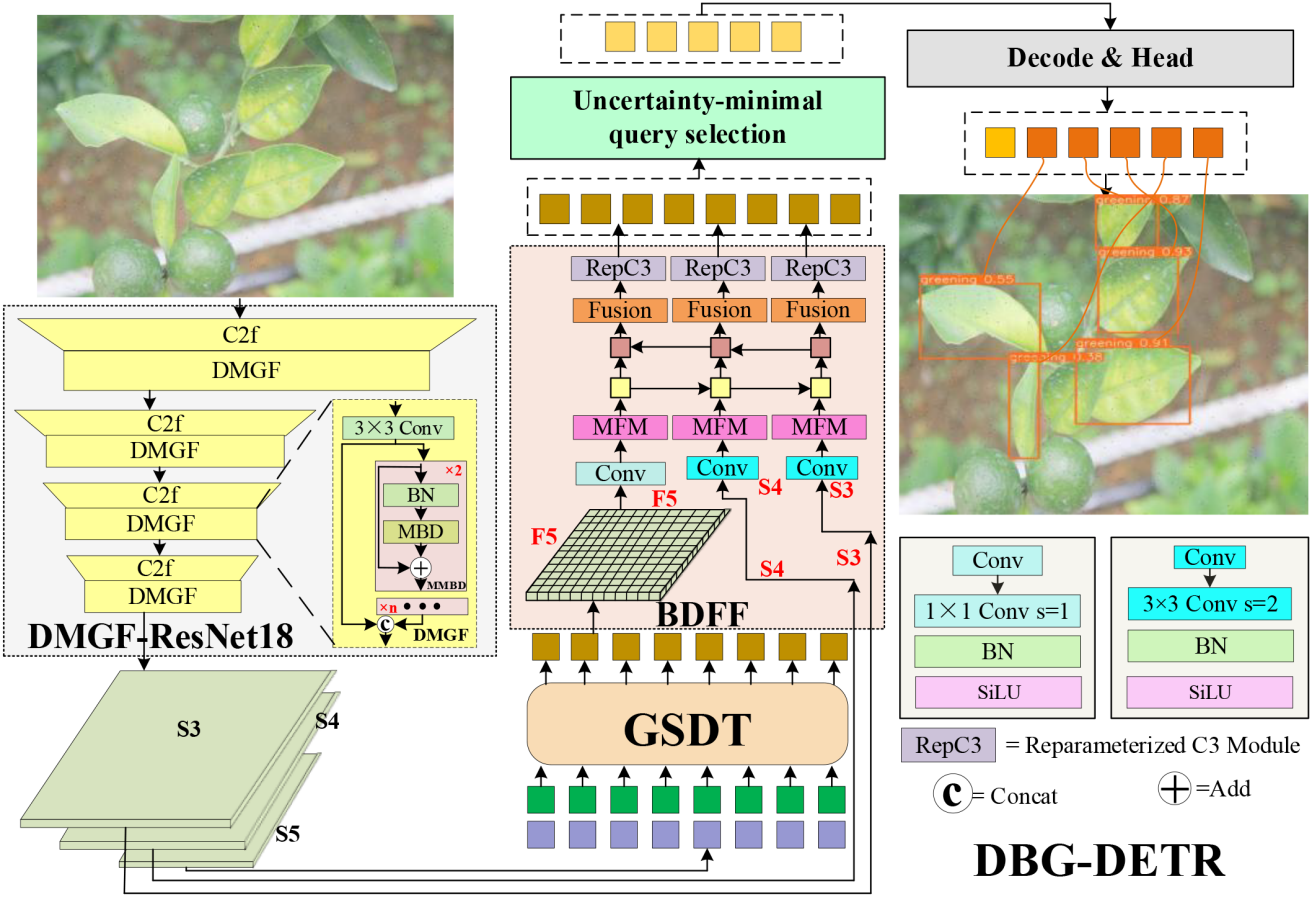
Overall architecture of the DBG-DETR network.

To address the limitations of RT-DETR, the overall architecture of the proposed DBG-DETR model is illustrated in **Figure 1**. First, input images of citrus diseased leaves are fed into a DMGF-ResNet18 backbone for feature extraction. The DMGF modules are embedded into the four C2f layers of ResNet18 to enhance feature representation for small-scale and occluded disease regions. The backbone outputs the last three feature maps, denoted as S3, S4, and S5, among which S5 contains the richest semantic information. Then, the S5 feature map is input into the GSDT module, which employs dynamic gating and a Top-K selection mechanism to adaptively assign higher attention weights to small and occluded disease regions. This process facilitates complementary feature enhancement and information reconstruction for small-target and occluded diseases, yielding an enhanced feature representation F5. Subsequently, F5, together with S3 and S4, is fed into the BDFF module. Through a bidirectional densely connected multi-scale pyramid structure, BDFF enables deep cross-level feature interaction and fusion, effectively alleviating detail loss and feature dilution during feature sampling. As a result, the model’s robustness in identifying and localizing diseases under occlusion, illumination variation, and diverse morphological patterns in complex orchard environments is substantially improved. Finally, the fused features are processed by the uncertainty-minimal query selection mechanism and the detection head to generate the final disease detection results.

### 3.1 DMGF-ResNet18

**Figure 2:**
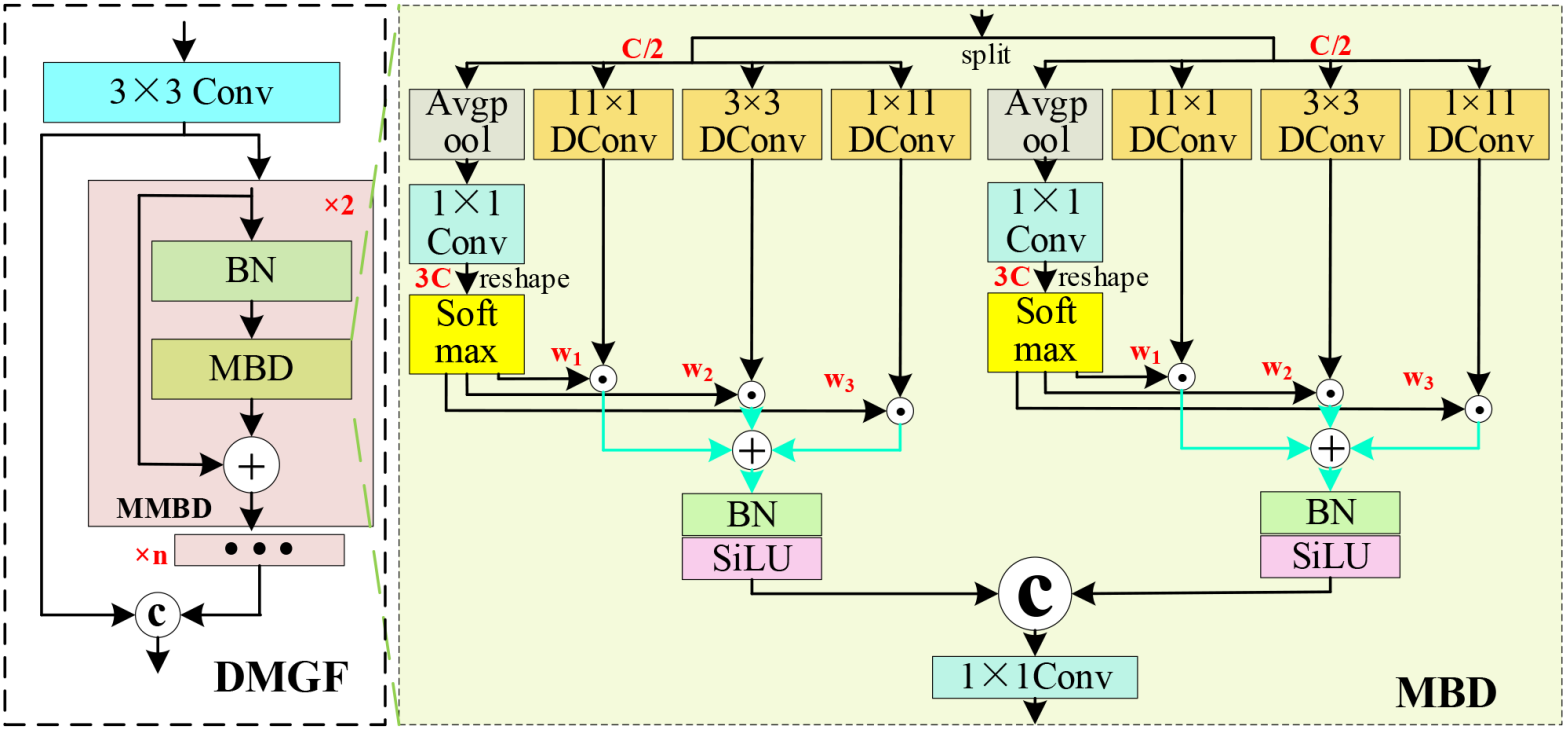
Architecture of the MBD network.

The baseline RT-DETR employs the multilayer stacked convolutional and pooling architecture of ResNet-18 to extract features of leaf diseases. However, during repeated downsampling through convolution and pooling operations, subtle features associated with small-scale disease targets are easily lost, thereby increasing the risk of missed detections. To address this limitation, a DMGF-ResNet18 network is proposed, as illustrated in **Figure 1**. In this network, DMGF modules are embedded into the four original C2f layers of ResNet18 to enhance feature extraction for small-scale and occluded disease regions, thereby enabling the acquisition of more complete and discriminative multi-scale disease representations. The architecture of the DMGF module is illustrated in **Figure 2**. Specifically, the input feature *X* is first processed by a 3×3 convolution layer to adjust channel dimensions and spatial scales. The adjusted feature is then fed into a multilayer MMBD (multi-layer MBD) structure, where it is fused with the input feature map via skip connections and concatenation. Finally, the output feature map is generated through MMBD-based feature computation. The MMBD branch is constructed by cascading MBD submodules with identical structures. Within each MBD submodule, feature reuse and enhancement are achieved through batch normalization and skip connections in the MBD module, thereby further improving the feature representation capability.

The network structure of the MBD submodule is illustrated in **Figure 2**. First, the input feature is split into two parts along the channel dimension. The split features are then processed by three parallel DConv with kernel sizes of 11×1, 1×11 and 3×3, which respectively capture long range vertical, long range horizontal, and local neighborhood information. This design is motivated by prior knowledge of disease morphology. After channel-wise splitting, the parallel branches are used to extract vertical leaf-vein textures, horizontally extended contextual information, and local fine-grained lesion features. Compared with dilated convolution, the dense sampling strategy better preserves lesion integrity. Compared with asymmetric convolution, the parallel design enables more complete capture of leaf-vein-related features while reducing feature aliasing. Subsequently, the vertical, horizontal, and local features are passed through average pooling, 1×1 convolution, and Softmax operations to compute their respective weights, which are used for weighted feature fusion. Finally, the features from the two branches are concatenated and fed into a 1×1 convolution to adjust the channel dimensions, producing the final MBD enhanced feature map. The MBD submodule adopts a parallel dual-branch architecture, enabling synchronized parallel computation to improve computational efficiency while capturing structural and pathological features across different orientations. This design facilitates fine grained discrimination of disease textures along leaf veins, horizontally spreading lesions, and localized pathological regions.

The DMGF module computes the input feature *X* according to **Equation 1**.

(1) $$\begin{array}{c} F_{\text{DMGF}}= \end{array}[f_{cov3\times 3};F_{\text{MMBD}}(f_{cov3\times 3}(X))]$$

where $F_{\text{DMGF}}$ denotes the output feature of the DMGF module, $f_{cov3\times 3}$ represents the 3×3 convolution, $F_{\text{MMBD}}$ denotes the output feature of MMBD, and []; indicates the concatenation. The MBD module computes the input feature *X* according to **Equation 2**.

(2) $$\begin{array}{c} F_{\text{MBD}}=f_{cov1\times 1}[\begin{array}{c} \alpha (B_{\text{norm}}(w_{1}f_{Dcov11\times 1}(X_{c/2})+w_{2}f_{Dcov1\times 11}(X_{c/2})+w_{3}f_{Dcov3\times 3}(X_{c/2})));\\ \alpha (B_{\text{norm}}(w_{1}f_{Dcov11\times 1}(X_{1-c/2})+w_{2}f_{Dcov1\times 11}(X_{1-c/2})+w_{3}f_{Dcov3\times 3}(X_{1-c/2}))) \end{array}] \end{array}$$

where $f_{Dcov11\times 1}$ denotes the DConv with a kernel size of 11×1, *w* represents the weights of vertical, horizontal, and local features computed via average pooling, 1×1 convolution, and Softmax, $B_{\text{norm}}$ denotes the batch normalization (BN), and *α* represents the SiLU activation function.

### 3.2 BDFF

**Figure 3:**
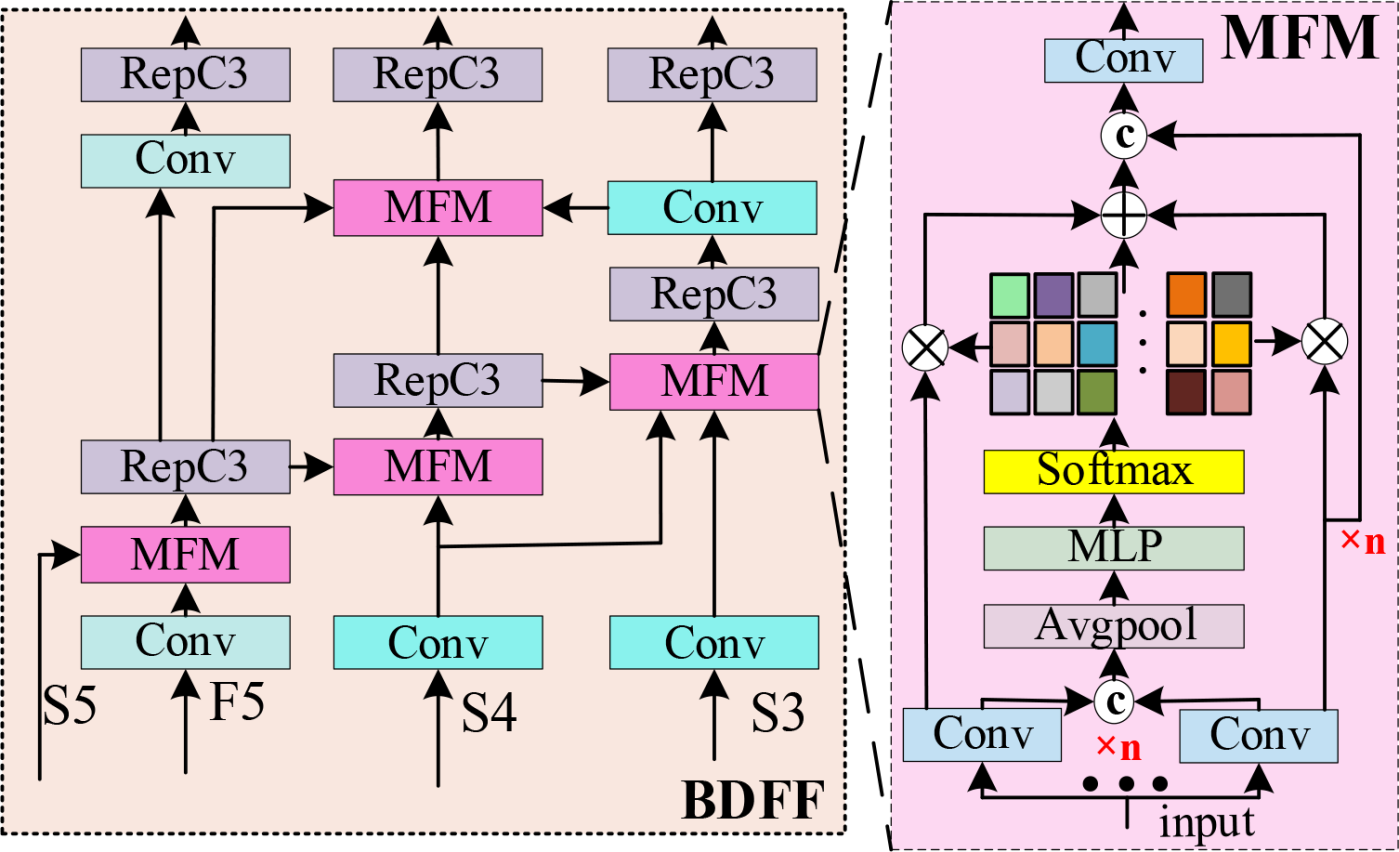
Architecture of the BDFF network.

To address feature degradation and background interference caused by leaf occlusion, uneven illumination, and diverse disease morphologies in orchard environments, as well as detail loss and feature sparsity during feature sampling and computation, a BDFF module is designed, as illustrated in **Figure 3**, inspired by the MFM (Zhang et al., 2024) and FPN (Feature Pyramid Network) (Li and Liu, 2025) The BDFF module achieves cross-level feature interaction through a bidirectional feature pyramid structure and further incorporates an adaptive feature selection mechanism, in which deep semantic information guides the enhancement of shallow details, while shallow texture cues compensate for the localization boundaries of deep features. In this way, the representation capability of multi-scale features is strengthened in a coordinated manner. This mechanism enables the model to focus more effectively on disease regions while suppressing background interference, thereby significantly improving the detection performance and localization accuracy of citrus leaf diseases in complex background environments.

The BDFF module takes four feature maps S5, F5, S4, and S3, as inputs, as illustrated in **Figure 3**. First, the shallow features S4 and S3 are processed by 3×3 convolution for downsampling and channel alignment, to facilitate subsequent cross scale feature fusion. The deep feature S5 and the GSDT enhanced feature F5 are then fed into MFM for weighted feature fusion. Subsequently, the aligned shallow features and the fused deep features are respectively passed through the MFM module to enable feature interaction, after which the interacted features are further processed by the RepC3 modules for feature enhancement. Finally, through progressive processing by three RepC3 layers, fused multi-scale feature representations are obtained. The structure of the MFM module is illustrated in **Figure 3**. This module is designed to flexibly handle multiple input feature streams and perform adaptive feature fusion. Taking S5 and F5 as an example, the MFM module first applies a 1×1 convolution to adjust the channel dimensions of each input feature. The adjusted features are then divided into three branches, including two side branches and one middle branch. The two side branches, denoted as $f_{cov1\times 1}(S5)$ and $f_{cov1\times 1}(F5)$, are used to preserve the original feature information, while the middle branch learns fusion weights from the concatenated features. These learned weights are subsequently applied to the side-branch features to perform weighted feature fusion, thereby achieving effective cross-level feature interaction and integration.

The computation of the MFM module for the S5 and F5 features is formulated according to **Equations 3**, **4**.

(3) $$\begin{array}{c} w_{i}\;(i=1,2)=f_{\text{avgpool}}(f_{\text{MLP}}(\beta [f_{cov1\times 1}(S5);f_{cov1\times 1}(F5)])) \end{array}$$

(4) $$\begin{array}{c} F_{\text{MFM}}=f_{cov1\times 1}(w_{1}\cdot f_{cov1\times 1}(S5)+w_{2}\cdot f_{cov1\times 1}(F5)) \end{array}$$

where $f_{\text{avgpool}}$ denotes the global average pooling, $f_{\text{MLP}}$ represents the MLP computation, β denotes the Softmax function, $f_{cov1\times 1}$ represents the 1×1 convolution, $F_{\text{MFM}}$ denotes the output feature of the MFM module, and $w_{1}$ represents the computed feature weights for S5 or F5.

### 3.3 GSDT

The baseline RT-DETR enhances deep feature S5 using a self-attention mechanism, producing the enhanced feature representation F5. However, self-attention (Gao et al., 2022) requires separate computation of the key K, query Q, and value V matrices, and relies on computationally intensive matrix multiplication operations to model the interactions and enhancement among K, Q and V, Its computational complexity is ${\left(H\times W\right)}^{2}d$, which significantly increases the computational burden of the network. In addition, self-attention performs dense global encoding over all spatial positions, inevitably mixing discriminative disease features with a large amount of background information. This results in feature redundancy and ambiguity, thereby degrading detection accuracy in complex orchard environments. Inspired by the efficient prompt guide operator (EPGO) (Wu et al., 2025), an adaptive intra-scale feature interaction module is designed for GSDT. This module replaces the matrix multiplication operations in conventional self-attention with a linear attention mechanism, reducing the computational complexity from quadratic to linear order, namely H×W×d+H×W×K×d, where *K* denotes the number of key points selected by Top-K. As a result, the resource overhead during network inference is substantially reduced. On this basis, a dynamic gating mechanism is introduced to adaptively modulate channel-wise feature responses, while Top-K sparse attention is employed to identify critical disease regions in the spatial dimension. This enables the model to focus on the core lesion regions of leaves while effectively suppressing background interference. Through this collaborative design, the proposed module reduces computational burden while precisely enhancing the model’s discriminative capability for citrus leaf disease features, thereby providing a feasible solution for real-time deployment on edge devices.

**Figure 4:**
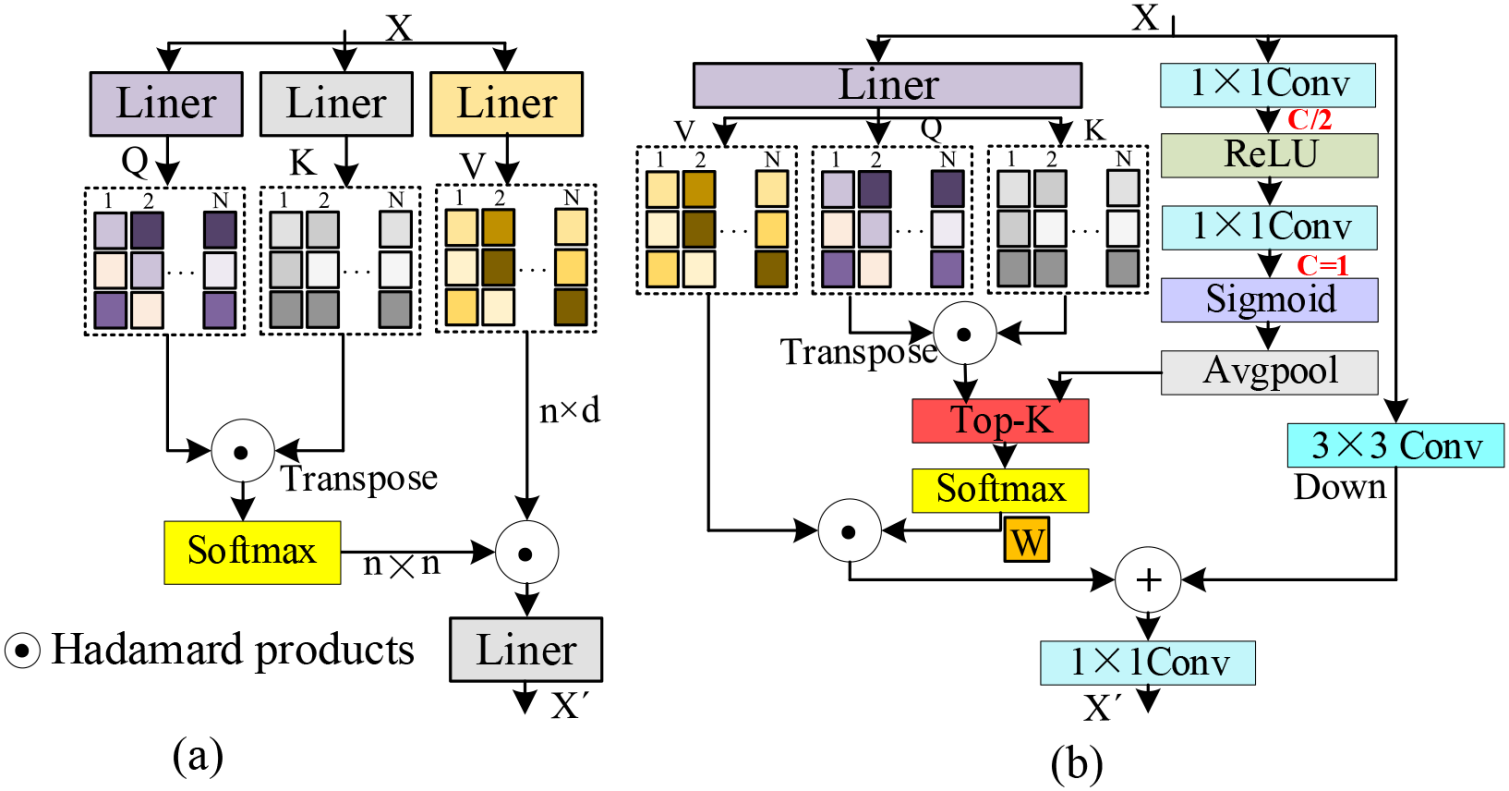
Network architectures of self-attention and DGST. **(a)** Self-attention. **(b)** DGST.

As shown in **Figures 4a**, self-attention maps the input feature *X* into the K, Q and V matrices. The weight matrices along the K and Q directions are then multiplied and subsequently fused with the matrix along the V direction, yielding the self-attention-enhanced output feature according to **Equation 5**.

(5) $$\begin{array}{c} F_{\text{self-attention}}=\beta (\frac{QK^{\text{T}}}{\sqrt{d}})⊙V \end{array}$$

where *d* denotes the number of tokens, and β represents the Softmax activation function.

As illustrated in **Figure 4b**, the GSDT module achieves efficient feature interaction and enhancement through the fusion of three parallel branches. The first branch is responsible for computing the K, Q and V projection matrices, thereby establishing preliminary feature associations. The second branch is designed to generate dynamic sparse gating weights. Specifically, the input feature is first processed by a 3×3 convolution to reduce channel dimensions, followed by a ReLU activation, another 3×3 convolution, a Sigmoid function, and global average pooling, resulting in a one-dimensional gating vector. This vector enables the model to adaptively regulate attention density according to the input content. The third branch performs local downsampling, where a 3×3 convolution to extract and preserve local detail and positional information. During the attention sparsification stage, the one-dimensional gating vector generated by the second branch is used to perform Top-K selection on the attention weights produced by the first branch, retaining only the most informative feature connections to improve computational efficiency. Finally, the sparsified global contextual features are fused with the local features extracted by the third branch via linear addition, and the fused representation is passed through a 1×1 convolutional layer to adjust channel dimensions, yielding the aggregated feature output of the GSDT module. This design substantially reduces computational complexity while strengthening feature focus on critical disease regions.

In the first branch, the K, Q and V projection matrices are respectively computed from the input feature X according to **Equation 6**.

(6) $$\begin{array}{c} Q,K,V=S_{\text{plit}}(f_{cov1\times 1}(X)) \end{array}$$

where Q,K∈, $V\in {\mathbb{R}}^{B\times h\times N\times d_{v}}$, *h* denotes the number of attention heads, $d_{k}$ and $d_{v}$ represent the dimension of each attention head for the corresponding matrices. The second branch computes the dynamic sparsity gating weight $w_{\text{GDST}}$ according to **Equation 7**.

(7) $$\begin{array}{c} w_{\text{GDST}}=f_{\text{avgpool}}(\delta (f_{cov1\times 1}(\sigma (f_{cov1\times 1}(X))))) \end{array}$$

Here, $w_{\text{GDST}}\in {\mathbb{R}}^{B\times 1\times H\times W}$, δ denotes the Sigmoid activation function, σ represents the ReLU activation function, and $f_{\text{avgpool}}$ denotes the average pooling operation. The third branch performs dynamic Top-K attention, in which the gating weight $w_{\text{GDST}}$ generated by the second branch is computed according to **Equation 8**.

(8) $$\begin{array}{c} w_{\text{GDST}}^{'}=\beta (w_{\text{GDST}},T_{k}) \end{array}$$

$T_{k}$ denotes the Top-K gating computation, $T_{k}=G_{ate}(X)$, which is dynamically determined by the gating network based on the current input feature *X*. The output of the dynamic Top-K attention sparsification is then computed according to **Equation 9**.

(9) $$\begin{array}{c} F=\beta (w_{\text{GDST}}^{'}((\frac{QK^{\text{T}}}{\sqrt{d}}))⊙V \end{array}$$

where ⊙ denotes the Hadamard product. Finally, the output feature of the GSDT module is computed according to **Equation 10**.

(10) $$\begin{array}{c} F_{\text{GDST}}=f_{cov1\times 1}(F+f_{cov3\times 3}(X)) \end{array}$$

## 4 Experiments and results analysis

### 4.1 Dataset description

In this study, a publicly available dataset, namely the Detection Dataset for Citrus Lesion Spot (CLS dataset) (Sharif et al., 2018), together with a self-constructed Jiangxi citrus leaf disease dataset, was used for experimental evaluation. The CLS dataset consists of two subsets for citrus fruit diseases and citrus leaf diseases. Specifically, the citrus leaf disease subset contains 670 images covering five categories: Black-spot, Canker, Greening, Healthy, and Melanose. All images were collected in outdoor environments in the Sargodha region of Pakistan, with an image size of 520×530 pixels and a resolution of 300 dpi. Because the images were acquired under complex background conditions, the dataset poses considerable challenges for accurate disease recognition. In addition, the disease samples exhibit distinct visual characteristics, and the diversity and complexity of the dataset provide a sound basis for evaluating model robustness. The Jiangxi citrus leaf disease dataset was collected from real orchard environments in Ganzhou City, Jiangxi Province, China. This dataset consists mainly of images captured in real orchard scenes, supplemented by a small number of images obtained through web crawling. As the largest production region for navel oranges and citrus crops nationwide, this area provides data with strong representativeness and practical relevance. Ganzhou is located in a subtropical hilly and mountainous region characterized by a warm and humid climate with abundant sunlight, and is internationally recognized as a major citrus production belt. The orchards are predominantly distributed on red-soil hilly slopes, offering favorable natural conditions for citrus cultivation. However, alongside the rapid development of the orchard industry, citrus crops in this region are highly susceptible to diseases such as huanglongbing, which pose a substantial risk of large-scale transmission. Therefore, conducting leaf disease detection research in this region is of significant importance for practical production guidance and effective disease management (Yu et al., 2024).

Based on field investigations, disease outbreaks in the studied region occur most frequently during the rainy season from May to September. Accordingly, data collection in this study was concentrated within this period to accurately analyze disease causes and support the development of effective prevention and control strategies. The constructed Jiangxi Citrus Leaf Disease Dataset contains five categories, namely Black-spot, Canker, Greening, Healthy, and Melanose. To evaluate the generalization ability of the proposed model, multiple influencing factors and complex background conditions were deliberately considered during data acquisition. Specifically, in terms of imaging distance, the dataset includes close-range, mid-range, and long-range views. In terms of leaf morphology, the samples encompass frontal, backside, and sideview orientations. Regarding illumination conditions, various scenarios are covered, including front lighting, backlighting, and partial occlusion. Backgrounds incorporate natural elements such as sky, ground, and branches, as well as a limited number of manmade scenes. In addition, the dataset accounts for variations in object quantity, including both single-leaf targets and overlapping multi-leaf targets. As a result, the dataset integrates a wide range of common interference factors encountered in disease recognition tasks, providing a robust and reliable data foundation for model training and evaluation.

The collected Jiangxi Citrus Leaf Disease Dataset was first preprocessed by batch removal of blurred and low-quality images, particularly those caused by camera shake. Subsequently, manual screening was conducted to discard samples in which disease features were lost due to strong backlighting or targets were excessively occluded and therefore unrecognizable. To improve the generalization capability of the model, data augmentation techniques including random horizontal and vertical flipping, rotation within a specified angle range, and brightness adjustment were applied. All images were resized to 640×640, resulting in a citrus leaf disease dataset containing 5,658 images. The LabelMe tool (Russell et al., 2008) was employed for fine-grained annotation of disease targets, and corresponding TXT-format label files were generated according to disease categories. To ensure annotation consistency and accuracy, a set of standardized labeling guidelines was established. (1) Completeness: For leaves with clearly visible lesions, annotations should accurately and completely follow the true contours of the diseased regions. For systemic diseases such as greening, the entire leaf area should be annotated to ensure that all disease-related features are included. (2) Uniqueness: Annotated targets must be clearly distinguished from background interference. Regions affected by shadows or areas with colors similar to the background are excluded from disease annotations. (3) Consistency: In this study, all annotations were completed by a single annotator following the same set of labeling criteria, thereby ensuring annotation consistency and minimizing human-induced variability to the greatest extent possible. (4) Independence: When multiple diseased leaves appear in a single image, each leaf should be annotated as an independent target, regardless of occlusion or overlap with other leaves or objects. (5) Accuracy: All annotations must undergo systematic verification and correction. Label files are required to follow a unified format and standardized naming scheme, and must accurately correspond to the associated images to ensure data quality and reliability.

**Figure 5:**
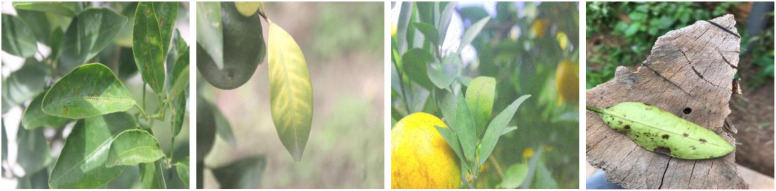
Examples of the dataset.

Finally, the two datasets were randomly divided into training, validation, and test sets at a ratio of 8:1:1. For the CLS dataset, the training set contained 536 images, while the validation and test sets each contained 67 images. For the Jiangxi Citrus Leaf Disease Dataset, the training set contained 4,526 images, while the validation and test sets each contained 566 images. Representative samples are shown in **Figure 5**.

**Figure 6:**
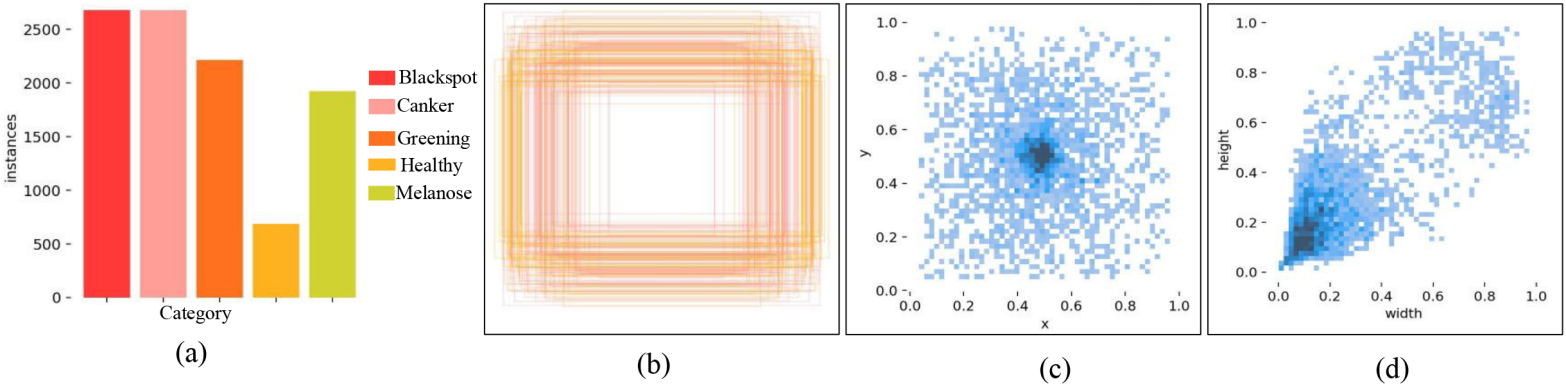
Overall label distribution of the dataset. **(a)** Number of tags, **(b)** Position of detection box, **(c)** Detection box size, **(d)** Relative size of detection box.

During dataset construction, the complexity of real world scenarios was fully considered, achieving comprehensive coverage across spatial scales, leaf morphologies, illumination conditions, and background environments, thereby providing a reliable foundation for model training and evaluation. The distribution of sample quantities for each category, as illustrated in **Figure 6a**, indicates that the dataset reflects practical application scenarios in which diseased samples are more prevalent. The spatial distribution of bounding box locations shown in **Figure 6b** reveals that most targets are densely concentrated in the central regions of the images, reflecting a focused and purposeful data acquisition strategy. Furthermore, the visualizations of absolute and relative target sizes in **Figures 6c, d** demonstrate that the majority of annotated regions in the dataset are small. This characteristic closely corresponds to the core challenge of detecting subtle features in early-stage disease identification and poses a meaningful challenge for evaluating the model’s ability to extract and localize small-scale and occluded targets.

### 4.2 Experimental environment and evaluation indicators

To verify the effectiveness of the proposed DBG-DETR model in leaf disease detection tasks, all models were trained and evaluated under identical experimental settings. The experiments were conducted using PyTorch 2.3.1 with Python 3.8.19. The hardware configuration included an Intel Core i5-14600KF CPU and an NVIDIA GeForce RTX 4080S GPU. The input image resolution was fixed at 640×640 pixels.

To ensure the rigor of the experimental design and the fairness of inter-model comparisons, all experiments in this study adopted a unified training-from-scratch strategy without loading pretrained weights. This decision was made because incompatibilities may arise between pretrained weights and the backbone architectures or newly introduced modules of certain comparison models. Under such circumstances, loading pretrained weights may lead to training instability and interfere with fair evaluation across different models. A unified training starting point can effectively eliminate prior bias introduced by differences in weight compatibility, thereby ensuring fair comparison under completely equivalent initial conditions. Furthermore, for the specific task of citrus leaf disease detection, removing pretrained weights helps reduce inductive bias inherited from classification tasks and prevents performance gains brought by external data transfer from masking the model’s intrinsic ability to learn task-specific feature representations from scratch. All models were optimized using the Adam optimizer, with a batch size of 4, 4 workers, 200 epochs, a non-maximum suppression (NMS) IoU threshold of 0.5, and a confidence threshold of 0.01.

To comprehensively evaluate the effectiveness of the proposed method, performance is assessed from three perspectives: detection accuracy, model complexity, and real-time performance. Detection accuracy is quantified using Precision (P), Average Precision (AP), mean Average Precision (mAP), mAP@[0.5:0.95] (mmAP), Recall (R), and F1-score (F1). Higher values of these metrics indicate better detection performance, corresponding to fewer false positives and missed detections. Model complexity is evaluated in terms of the number of parameters (Params) and giga floating-point operations (GFLOPs), where lower values indicate a more lightweight architecture. For real-time performance, a model is considered to satisfy real-time detection requirements when its frames per second (FPS) is at least 30, and higher FPS values indicate stronger real-time inference capability.

### 4.3 Ablation experiments

To verify the effectiveness of different improvement strategies in the DBG-DETR framework, ablation experiments were conducted on the same dataset under identical experimental settings. The variant incorporating DMGF-ResNet18 is denoted as M1, the variant equipped with the GSDT module is denoted as M2, and the variant employing the BDFF module is denoted as M3. The results are summarized in **Table 1**.

**Table 1:** Ablation comparison of evaluation metrics for each category.

Method	M1	M2	M3	mAP/%	mmAP/%	R/%	Para/M	GFLOPs	FPS frame/s
RT-DETR				95.71	85.13	94.43	20.09	58.34	85.71
	✓			97.14	86.55	96.18	13.42	44.90	93.84
	✓	✓		97.88	86.89	96.77	**13.38**	**44.44**	**99.19**
	✓	✓	✓	**99.11**	**89.24**	**98.32**	16.31	45.98	89.97

Bold content represents the best result of the indicator.

The results presented in **Table 1** demonstrate that each targeted optimization strategy contributes positively to citrus leaf disease detection. Compared with the baseline RT-DETR, DMGF-ResNet18 improves P by 1.79%, mAP by 1.43%, mmAP by 1.42%, R by 1.75%, and F1 by 1.77%, while reducing the number of parameters by 6.67 M and computational complexity by 13.44 GFLOPs, and increasing inference speed by 8.13 FPS. These results indicate that the DMGF module, by introducing DConv and jointly extracting vertical, horizontal, and local neighborhood features, effectively captures disease-related characteristics such as leaf-vein orientation, structural extension patterns, and local texture details. As a result, it enables more comprehensive disease feature extraction while simultaneously reducing model parameters and computational complexity. Building upon DMGF-ResNet18, the GSDT strategy further improves P by 1.79%, mAP by 0.74%, mmAP by 0.34%, R by 0.59%, and F1 by 1.77%, while reducing Params by 0.04 M and GFLOPs by 0.46, and increasing FPS by 5.35. These additional gains suggest that GSDT further enhances feature discrimination. By incorporating linear additive operations, dynamic gating, and a Top-K sparse attention mechanism, GSDT enables the network to focus more effectively on disease features in critical regions while suppressing interference from complex backgrounds. The BDFF module further improves P, mAP, mmAP, R, and F1 by 1.32%, 1.23%, 2.35%, 1.55%, and 1.53%, respectively, through its cross-level bidirectional feature interaction design. Although the number of parameters increases by 2.93 M, the overall model still satisfies the requirements for real-time inference and lightweight deployment. Overall, the proposed optimization strategies, including DMGF-ResNet18, GSDT, and BDFF, consistently improve both detection accuracy and computational efficiency for citrus leaf disease detection. Compared with the baseline RT-DETR, the final DBG-DETR model achieves improvements of 3.31% in P, 3.40% in mAP, 4.11% in mmAP, 3.89% in R, and 3.59% in F1, while reducing the number of parameters by 3.78 M and GFLOPs by 12.36, and increasing inference speed by 4.26 FPS.

**Figure 7:**
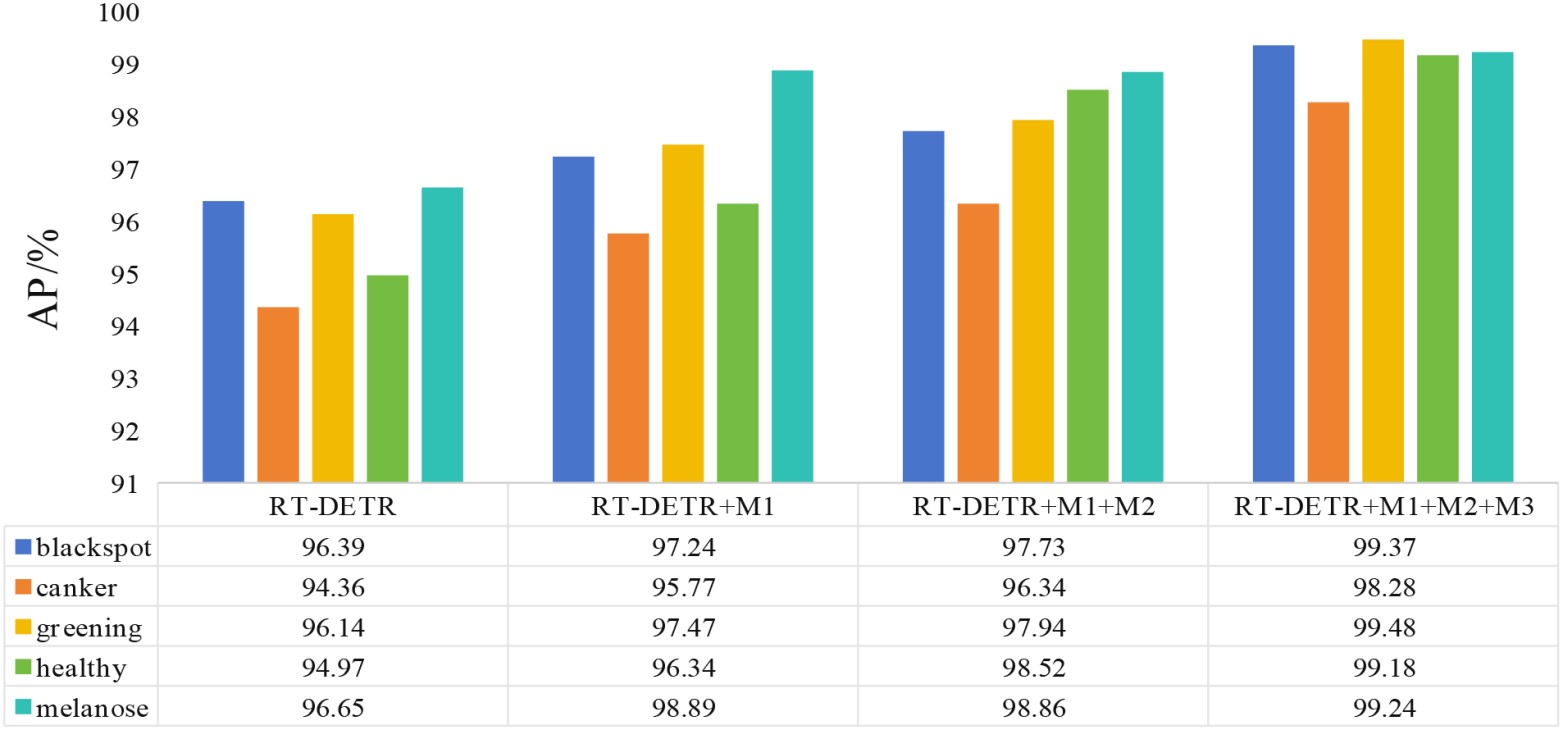
Ablation results of AP metrics for each category.

**Figure 7** presents the AP comparison of different optimization strategies across five categories: Black Spot, Canker, Greening, Healthy, and Melanose. The experimental results show that the three proposed optimization strategies, namely DMGF-ResNet18, GSDT, and BDFF, consistently improve AP for all categories when applied individually or in combination. Among these categories, the most significant improvements are observed for Black Spot and Melanose, with AP gains of 2.8% and 3.2%, respectively. These results demonstrate the effectiveness of DMGF-ResNet18 in enhancing multi-scale feature extraction within the backbone, the advantage of GSDT in modeling spatial contextual information, and the synergistic role of BDFF in fusing multi-level features, thereby improving the discrimination of complex and subtle disease patterns. For Canker and Greening, whose symptoms are relatively subtle and more easily confused with healthy leaves, AP also achieves stable improvements of 2.1% and 1.9%, respectively, further validating the effectiveness of the proposed method for detecting subtle and complex disease categories. Overall, the three optimization strategies achieve the best AP performance across all categories and are effective for both prominent and subtle lesions, thereby fully demonstrating the robustness and superiority of the proposed method in complex background environments and subtle lesion scenarios.

**Figure 8:**
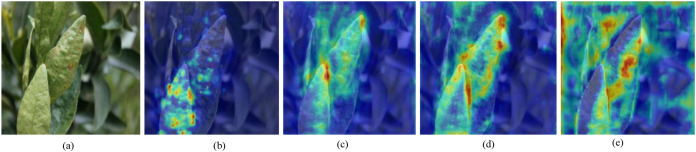
Heatmap visualization of the ablation study. **(a)** Original image, **(b)** RT-DETR, **(c)** RT-DETR+M1, **(d)** RT-DETR+M1+M2, **(e)** RT-DETR+M1+M2+M3.

To intuitively analyze the ability of each improvement module to focus on and represent disease features in complex orchard scenes, feature heatmaps are employed to visualize the distribution of model attention, as shown in **Figure 8**.The baseline RT-DETR model exhibits relatively dispersed attention over disease regions and demonstrates weak responses to leaf vein details and overlapping leaf areas. After introducing the DMGF-ResNet18 module, the effective receptive field of the network with respect to disease features is further expanded. However, the model still exhibits excessive sensitivity to adjacent non-diseased regions, which may lead to missed detections when handling occluded targets. With the incorporation of the GSDT module, the model’s attention becomes more concentrated on the overall contours of target leaves and local disease details, while interference from complex backgrounds is further suppressed. After integrating the BDFF module for cross-level feature fusion, the model is able to accurately extract and localize disease regions, significantly enhancing discrimination between diseased leaves and complex backgrounds and achieving precise attention focus on disease areas.

### 4.4 Comparative experiments

**Figure 9:**
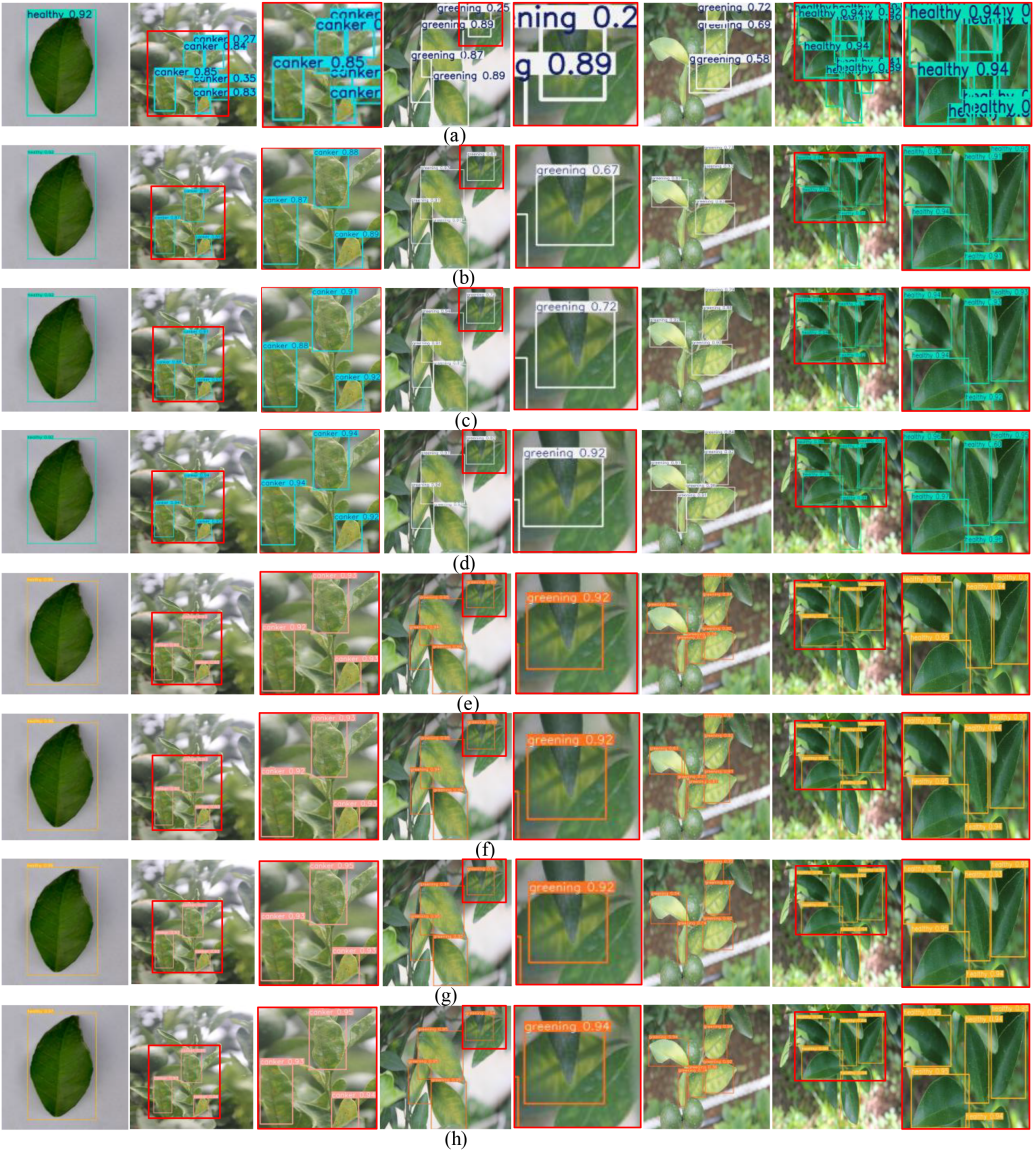
Visual results on the Jiangxi citrus leaf disease dataset. **(a)** Faster-RCNN, **(b)** YOULOv8M, **(c)** YOLOv111, **(d)** YOLOV121, **(e)** RT-DETR, **(f)** DINO, **(g)** MSHLB-DETR, **(h)** DBG-DETR.

**Figure 10:**
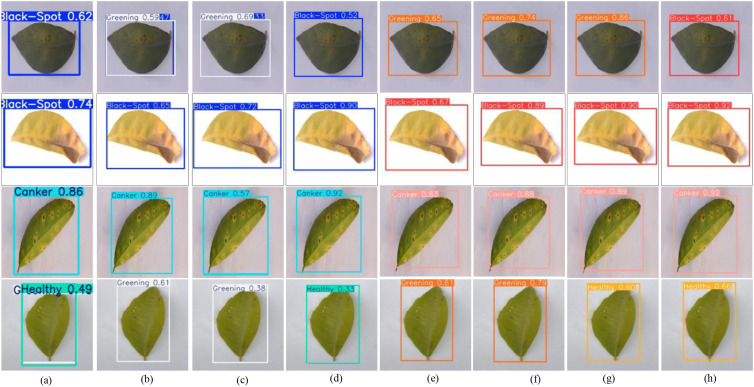
Visual detection results on the CLS dataset. **(a)** Faster-RCNN, **(b)** YOULOv8M, **(c)** YOLOv111, **(d)** YOLOV121, **(e)** RT-DETR, **(f)** DINO, **(g)** MSHLB-DETR, **(h)** DBG-DETR.

To verify the effectiveness of the proposed DBG-DETR method for leaf disease detection, comparative experiments were conducted on the CLS dataset and the Jiangxi Citrus Leaf Disease Dataset. The proposed method was compared with several widely used object detection algorithms, including Faster R-CNN (Cai et al., 2023), YOLOv8m (Liu et al., 2025a), YOLOv11l (Khanam and Hussain, 2024), YOLOv12l (Tian et al., 2025), RT-DETR (Zhao et al., 2023), and DINO (Caron et al., 2021). In addition, a specialized citrus leaf disease detection method, MSHLB-DETR (Liu et al., 2025b), was also included for comparison. The experimental results are presented in **Table 2**, **Figures 9**, **10**. , .

**Table 2:** Experimental comparison results of different methods.

Dataset	Methods	P/%	mAP/%	mmAP/%	R/%	F1/%	Para/M	GFLOPs	FPS frame/s
Jiangxi Citrus Leaf Disease Dataset	Faster-RCNN	91.29	90.58	82.82	92.38	91.98	54.12	93.33	28.42
	YOLOv8m	95.27	94.96	85.91	95.72	95.49	25.91	78.91	61.04
	YOLOv11l	97.05	96.47	86.27	96.47	96.76	25.37	86.88	63.15
	YOLOv12l	97.83	97.53	86.86	96.98	97.40	26.44	88.92	55.84
	RT-DETR	96.16	95.71	85.13	94.43	95.29	20.09	58.34	85.71
	DINO	98.69	98.33	87.93	97.84	98.26	22.24	91.70	73.24
	MSHLB-DETR	98.01	97.84	87.26	97.41	97.71	16.44	48.31	88.89
	DBG-DETR	**99.47**	**99.11**	**89.24**	**98.32**	**98.88**	**16.31**	**45.98**	**89.97**
CLS dataset	Faster-RCNN	87.77	74.87	63.91	77.76	82.46	54.12	93.33	28.42
	YOLOv8m	92.49	78.04	66.47	78.79	85.11	25.91	78.91	61.04
	YOLOv11l	97.56	82.26	68.60	84.28	90.46	25.37	86.88	63.15
	YOLOv12l	93.84	78.35	67.80	79.76	86.24	26.44	88.92	55.84
	RT-DETR	97.43	81.80	68.66	83.17	89.77	20.09	58.34	85.71
	DINO	98.79	83.44	68.73	82.93	90.18	22.24	91.70	73.24
	MSHLB-DETR	97.56	84.26	68.16	84.34	90.48	16.44	48.31	88.89
	DBG-DETR	**98.88**	**86.04**	**70.00**	**86.95**	**92.54**	**16.31**	**45.98**	**89.97**

Bold content represents the best result of the indicator.

**Table 2** shows that DBG-DETR achieves strong detection performance across multiple evaluation metrics on both datasets. Since the same comparison models were used on both datasets, the values of Params, GFLOPs, and FPS are identical for each algorithm across the two experimental settings. On the Jiangxi Citrus Leaf Disease Dataset, DBG-DETR achieves P, mAP, mmAP, R, and F1 values of 99.47%, 99.11%, 89.24%, 98.32%, and 98.88%, respectively. In addition, DBG-DETR requires only 16.31 M parameters and 45.98 GFLOPs, significantly improving detection speed while maintaining low computational cost, thereby achieving an effective balance between accuracy and efficiency. In contrast, the two-stage detector Faster R-CNN achieves only 28.42 FPS, making it difficult to satisfy real-time detection requirements. Compared with the specialized citrus disease detection model MSHLB-DETR, DBG-DETR attains better results in terms of mAP, mmAP, R, and FPS, while maintaining lower parameter count and computational cost. On the CLS dataset, DBG-DETR also achieves the best performance in terms of P, mAP, mmAP, R, and F1, reaching 98.88%, 86.04%, 70.00%, 86.95%, and 92.54%, respectively, which demonstrates its generalization capability across different citrus leaf disease datasets. Overall, these comparative results demonstrate that DBG-DETR has stronger feature extraction capability and robustness for leaf disease detection in complex orchard scenarios characterized by uneven illumination, overlapping branches and leaves, and small lesion regions, thereby validating the effectiveness, soundness, and generalization ability of the proposed network architecture. **Figure 9** illustrates the visual detection performance of different models under various real world scenarios on the Jiangxi Citrus Leaf Disease Dataset. The first column shows a simple background scenario containing a single diseased leaf. The second column presents a complex background scenario, and the third column provides a local magnified view of the second column. The fourth column corresponds to scenarios with occluded targets, while the fifth column shows the associated local magnifications. The sixth column represents dense-target scenarios, and the seventh column presents cases with overlapping branches and leaves.

The eighth column provides a locally magnified detail view of the seventh column. In the simple background scenario shown in the first column, all comparison algorithms, including Faster R-CNN, YOLOv8m, YOLOv11l, YOLOv12l, RT-DETR, DINO, MSHLB-DETR, and DBG- DETR, are able to accurately detect healthy leaves. However, DBG-DETR achieves the highest detection accuracy of 97%. In the complex background scenario shown in the second column, Faster R-CNN exhibits evident false detections, characterized by overlapping bounding boxes and multiple predictions for the same target. In contrast, the other comparison methods are able to correctly detect the diseased leaves, and the locally magnified results in the third column further indicate that DBG-DETR achieves the highest detection precision. In the occluded target scenario shown in the fourth column, Faster R-CNN suffers from substantial localization errors. As further illustrated by the magnified views in the fifth column, DBG-DETR demonstrates superior detection performance for occluded targets. In the dense target scenario shown in the sixth column, Faster R-CNN exhibits severe false positives and missed detections due to extensive bounding box overlap. YOLOv8m, YOLOv11l, YOLOv12l, and MSHLB-DETR exhibit missed detections, while DINO generates false detections caused by overlapping - predictions. Overall, the qualitative comparison results indicate that Faster R-CNN performs poorly in citrus leaf disease detection, whereas baseline detectors such as YOLOv8m, YOLOv11l, YOLOv12l, RT-DETR, and DINO perform well in simple scenarios but still exhibit limitations in complex, occluded, and dense scenes. The proposed DBG-DETR consistently demonstrates stable and efficient detection performance across all five scenario types, satisfying both accuracy and real-time requirements for leaf disease detection in practical orchard environments.

**Figure 10** presents the visual detection results on the public CLS dataset. As can be observed, Faster R-CNN exhibits overlapping false detections in the fourth row. YOLOv8m shows overlapping false detections in the first row and false detections in the fourth row. YOLOv11l also produces false detections in both the first and fourth rows. By contrast, YOLOv12l correctly detects the citrus leaf disease regions in all displayed cases. RT-DETR and DINO both exhibit false detections in the first and fourth rows, whereas MSHLB-DETR shows false detections in the first row. In comparison, the proposed DBG-DETR produces neither false detections nor missed detections in these examples. Moreover, compared with YOLOv12l, DBG-DETR achieves higher detection precision. Overall, the visual results on the two datasets demonstrate that the proposed method can more accurately recognize citrus leaf diseases under different scenarios while effectively avoiding false detections and missed detections.

## 5 Conclusion

To improve the detection accuracy of citrus leaf diseases in complex scenarios, this study proposes a DBG-DETR leaf disease detection method. Firstly, a comprehensive citrus orchard leaf disease dataset is collected and constructed according to the characteristics of the study region. A DMGF-ResNet18 feature extraction strategy is then introduced to capture richer disease features, effectively alleviating the challenge of key disease information loss in small-scale and occluded targets. Secondly, a BDFF-based feature fusion method is proposed to mitigate missed detections caused by feature degradation and interference under occlusion conditions. Finally, a lightweight attention module, GSDT, is designed to enhance the model’s capability to distinguish disease regions from complex backgrounds in challenging environments. Ablation experiments verify the effectiveness of each proposed component. Compared with the baseline RT-DETR, the proposed DBG-DETR achieved improvements of 3.31% in P, 3.40% in mAP, 4.11% in mmAP, 3.89% in R, and 3.59% in F1 on the Jiangxi Citrus Leaf Disease Dataset, while reducing the number of parameters by 3.78 M and GFLOPs by 12.36, and increasing FPS by 4.26. On the CLS dataset, the proposed method further improved P by 1.45%, mAP by 4.24%, mmAP by 1.34%, R by 3.78%, and F1 by 2.77%. Comparative experiments further verified the effectiveness and superiority of the proposed method for citrus leaf disease detection, demonstrating that it provides an efficient and accurate solution for intelligent citrus disease monitoring. Nevertheless, the current method remains insufficient for robust feature extraction under dim or low-light conditions, where false detections and missed detections are more likely to occur, thus limiting its practical applicability in all weather orchard scenarios. Future research will focus on developing robust low-light recognition algorithms, potentially in combination with image enhancement techniques, to further improve model perception under adverse illumination. Meanwhile, the proposed method can be integrated into a multi-view orchard camera network and collaboratively deployed with an autonomous citrus-picking robot platform to establish an all weather, full coverage smart agricultural monitoring system. This would support real-time perception and precise management of crop growth status, diseases, insect pests, and related information, thereby providing reliable technical support for precision agriculture in orchards. 

## Data Availability

The original contributions presented in the study are included in the article/supplementary material. Further inquiries can be directed to the corresponding author.
